# A prospective trial comparing FDG-PET/CT and CT to assess tumor response to cetuximab in patients with incurable squamous cell carcinoma of the head and neck

**DOI:** 10.1002/cam4.294

**Published:** 2014-08-01

**Authors:** Douglas Adkins, Jessica Ley, Farrokh Dehdashti, Marilyn J Siegel, Tanya M Wildes, Loren Michel, Kathryn Trinkaus, Barry A Siegel

**Affiliations:** 1 Division of Medical Oncology, Department of Internal Medicine, Washington University School of MedicineSt. Louis, Missouri; 2 Alvin J. Siteman Cancer Center, Washington University School of MedicineSt. Louis, Missouri; 3 Division of Nuclear Medicine, Mallinckrodt Institute of Radiology, Washington University School of MedicineSt. Louis, Missouri; 4 Division of Biostatistics, Washington University School of MedicineSt. Louis, Missouri

**Keywords:** Cetuximab, CT, FDG-PET/CT, head and neck, squamous cell carcinoma

## Abstract

Computed tomography (CT), the standard method to assess tumor response to cetuximab in incurable squamous cell carcinoma of the head and neck (SCCHN), performs poorly as judged by the disparity between high disease control rate (46%) and short time to progression (TTP) (70 days). F-18 fluorodeoxyglucose positron emission tomography (FDG-PET)/CT is an alternative method to assess tumor response. The primary objective of this prospective trial was to evaluate the metabolic response of target lesions, assessed as the change in maximum standardized uptake value (SUV_max_) on FDG-PET/CT before and after 8 weeks (cycle 1) of cetuximab. Secondary objectives were to compare tumor response by CT (RECIST 1.0) and FDG-PET/CT (EORTC criteria) following cycle 1, and determine TTP with continued cetuximab administration in patients with disease control by CT after cycle 1 but stratified for disease control or progression by FDG-PET/CT. Among 27 patients, the mean percent change of SUV_max_ of target lesions after cycle 1 was −21% (range: +72% to −81%); by FDG-PET/CT, partial response (PR)/stable disease (SD) occurred in 15 patients (56%) and progression in 12 (44%), whereas by CT, PR/SD occurred in 20 (74%) and progression in 7 (26%). FDG-PET/CT and CT assessments were discordant in 14 patients (*P* = 0.0029) and had low agreement (*κ* = 0.30; 95% confidence interval [CI]: 0.12, 0.48). With disease control by CT after cycle 1, median TTP was 166 days (CI: 86, 217) if the FDG-PET/CT showed disease control and 105 days (CI: 66, 159) if the FDG-PET/CT showed progression (*P* < 0.0001). Median TTP of the seven patients whose post cycle 1 CT showed progression compared to the 12 whose FDG-PET/CT showed progression were similar (53 [CI: 49, 56] vs. 61 [CI: 50, 105] days, respectively). FDG-PET/CT may be better than CT in assessing benefit of cetuximab in incurable SCCHN.

## Introduction

Cetuximab, a monoclonal antibody directed against the epidermal growth factor receptor (EGFR), is the only targeted therapy approved for treatment of squamous cell carcinoma of the head and neck (SCCHN). Indications include use as a single agent in platinum-resistant disease [1], in combination with chemotherapy for incurable disease [2], or concurrently with radiation therapy for locally advanced disease [3]. Benefits of cetuximab include improvement in tumor response, disease control and overall survival (OS) [2, 3].

In incurable SCCHN, single-agent cetuximab resulted in a tumor response rate of 13%, disease control rate of 46%, and median time to progression (TTP) and OS of 70 and 178 days, respectively [1]. A small fraction of patients have progression-free survival for more than 6 months. Early identification of benefit from cetuximab is important, since efficacy overall is limited.

Computed tomography (CT) is the standard method for assessing tumor response to cetuximab in incurable SCCHN. However, it is unclear if CT is the optimal method, and it has not been prospectively compared to alternative imaging modalities. Currently, biomarkers that predict response of SCCHN to cetuximab do not exist. Evidence supports investigation of positron emission tomography (PET) with F-18 fluorodeoxyglucose (FDG)-PET/CT to evaluate tumor response to EGFR inhibitors. Changes in FDG uptake correlated with molecular response to the EGFR inhibitor erlotinib in SCCHN cell lines and xenograft models and in a pilot study of neoadjuvant erlotinib [4]. In a neoadjuvant trial of cetuximab given to patients with SCCHN, changes in FDG uptake correlated to declines in Ki-67 expression and tumor cellularity [5]. In selected malignancies, tumor response, TTP and OS with targeted therapy were better predicted by sequential FDG-PET/CT than by CT [6]. Several reviews highlighted the limitations of anatomic imaging (by CT) using response evaluation criteria in solid tumors (RECIST 1.0) [7], particularly with respect to targeted therapy, and noted the benefits of metabolic tumor response assessment with FDG-PET [8, 9].

In this prospective trial, we sought to compare tumor response assessment by CT and FDG-PET/CT following 8 weeks (cycle 1) of single-agent cetuximab administered to patients with incurable SCCHN. In addition, we determined TTP with continued cetuximab administration in patients with disease control by CT after cycle 1 but stratified for disease control or progression by FDG-PET/CT.

## Materials and Methods

### Patient selection

Eligible patients were 18 years of age and older with incurable (metastatic or unresectable locoregional recurrence in a previously irradiated field) SCCHN. At least one PET-measurable lesion was required, which we defined as a lesion ≥1.5 cm by CT that was FDG avid (maximum standardized uptake value [SUV_max_] ≥ 3) by FDG-PET/CT. Eastern Cooperative Oncology Group (ECOG) performance status of 0–3 was acceptable. Prior therapy with an EGFR-specific monoclonal antibody was allowed only if it was given as part of definitive treatment for nonmetastatic disease occurring more than 3 months beforehand. Exclusion criteria included cancer therapy received within 14 days and prior grade 4 hypersensitivity infusion reaction (HSR) to cetuximab. All study participants signed informed consent for this institutional review board-approved, prospective trial (Clinicaltrials.gov NCT#00671437).

### Treatment plan and standard assessments

Baseline assessments performed within 28 days of treatment included history taking and physical examination, contrast-enhanced CT of neck and chest and FDG-PET/CT. Cetuximab was given in 8 week cycles as a 400 mg/m^2^ loading dose IV followed by weekly doses of 250 mg/m^2^. One week (±3 days) after cycle 1, patients underwent assessment of tumor response with CT and FDG-PET/CT. Tumor response was assessed by CT using RECIST 1.0 [7] and by FDG-PET/CT using the European Organization for Research and Treatment of Cancer (EORTC) criteria [10]. In brief, definitions of metabolic response by FDG-PET/CT included: complete metabolic response (CMR)—complete resolution of all metabolically active target and nontarget lesions, and no new lesions; partial metabolic response (PMR)—20% or greater decrease in SUV of target lesions with or without decrease in number/size of nontarget lesions, and no new lesions; progressive metabolic disease (PMD)—one or more new lesions, 20% or greater increase in SUV of target lesions and/or unequivocal increase in FDG activity of nontarget lesions; and stable metabolic disease (SMD)—not qualifying as CMR, PMR, or PMD.

Evaluation of response by CT with RECIST 1.0 was performed by an independent radiologic reviewer (M. J. S.). Target lesions were chosen based on the size (≥1.0 cm) with inclusion of locoregional disease and distant metastases. Necrotic lesions were avoided. Patients with disease control (complete response [CR], partial response [PR] or stable disease [SD]) by RECIST 1.0 at end of cycle 1 continued on treatment with cetuximab until there was evidence of progressive disease by RECIST. CT scans were performed at the end of each cycle of cetuximab; however, FDG-PET/CT was performed only following cycle 1. Since little was known about the correlation of metabolic tumor response by FDG-PET/CT to anatomic tumor response by CT with respect to TTP in patients with SCCHN receiving cetuximab, the results of the FDG-PET/CT were not used to define disease progression. Decisions about whether or not to continue cetuximab after cycle 1 were based on the tumor response assessment by CT.

Noniodine contrast-enhanced FDG-PET/CT was performed with one of several PET/CT scanners (Siemens Biograph 40HD (Siemens Medical Solutions USA, Inc., Malvern, PA), Siemens mCT (Siemens Medical Solutions USA, Inc.), and GE Discovery STE, GE Healthcare, Waukesha, WI) before and following 8 weeks of cetuximab in accordance with the Division of Nuclear Medicine's standard procedures based on the National Cancer Institute recommendations [11]. Both scans were performed on the same model of scanner. The standard whole-body examination included images from the skull vertex to the upper thighs, acquired in two acquisitions. The first acquisition consisted of two bed positions from the skull vertex to the lung apices and the second acquisition encompassed the neck to the upper thighs. FDG, 10–15 mCi (dose adjusted up to 25 mCi for obese subjects), was administered IV and imaging was begun 60 ± 10 min later. CT images used for attenuation correction and image fusion were acquired at 120 kVp with 95–111 mAs. Emission scan duration ranged from 2 to 5 min per bed position, depending on body weight. Images were reconstructed at 5-mm slice thickness.

FDG-PET/CT images were evaluated qualitatively as well as quantitatively by one of two experienced nuclear radiologists (F. D., B. A. S.). For quantitative analysis, SUV_max_ within each of the tumor sites was determined within a volume of interest around the tumor using a Siemens eSoft workstation (Siemens Medical Solutions USA, Inc.). Up to a maximum of three target lesions (≥1.5 cm on the baseline CT) were identified as target lesions on the baseline FDG-PET. When multiple lesions were present, those having the greatest FDG uptake on the baseline FDG-PET were selected as target lesions. Lesions containing areas of necrosis were avoided. Other metabolically active lesions and lesions that were <1.5 cm on CT were considered nontarget lesions. When more than one target lesion was identified, the average percentage change in SUV_max_ was used to determine metabolic response. Target and nontarget lesions as defined above for the FDG-PET component of the study may or may not be the same target and nontarget lesions defined by RECIST 1.0. However, one or more of the target lesions identified on FDG-PET/CT were also identified as target lesions on the CT scan in 25 of the 27 evaluable patients.

### Statistical methods

#### Study objectives

The primary objective of the study was to compare the SUV_max_ of up to three target lesions as assessed by FDG-PET/CT performed before and after 8 weeks of cetuximab given to patients with incurable SCCHN. Secondary objectives included determining the overall tumor metabolic response (by FDG-PET/CT) and anatomic response (by CT) rates after 8 weeks of cetuximab. The results of tumor response as assessed by FDG-PET/CT and CT were compared and were correlated to TTP.

#### Data analysis

This was a single stage, nonrandomized, prospective observational study to determine the changes in SUV_max_ on FDG-PET/CT associated with 8 weeks of cetuximab therapy in patients with incurable SCCHN. For the primary endpoint, we determined the SUV_max_ for one to three target tumor sites (with the highest SUVs) in each patient at baseline and after cycle 1 of cetuximab. A clustered linear repeated measures model was used to compare one to three observations of SUV at two time points. SUV distribution was analyzed on a log scale to approximate a Gaussian distribution. Model fit was assessed using standardized and scaled residuals. Mean SUVs pre and post 8 weeks of cetuximab were estimated after adjusting for prior exposure to cetuximab, use of cetuximab as first-line therapy and tumor site (oropharynx vs. other). A generalization of McNemar's test was used to test for concordance of response (partial, stable or progression) by CT and by FDG-PET/CT. Kaplan–Meier and Cox proportional hazard models were used to describe the effects of metabolic and anatomic response (disease control or progression) on TTP. The proportional hazards assumption was examined using plots, and deviance and martingale residuals were examined to verify model fit.

#### Sample size and study power

Based on the institutional historical data, 40 new cases of incurable SCCHN were expected to present within the planned 2-year accrual period. Seventy percent, or 28 patients, were expected to (1) meet eligibility criteria, (2) consent to participate on the study, and (3) have measurable FDG uptake at tumor sites. Of those 28 patients, ∼3 were expected to discontinue therapy before the end of cycle 1 of cetuximab therapy because of adverse events, early disease progression, or other factors. The sample available for analysis was expected to be 25 patients. When the study was designed, little was known of the expected metabolic response distribution by SCCHN tumors obtained from patients at baseline and after cetuximab therapy. However, as an estimate, a sample of 25 patients provided at least 80% power at a 0.05 significance level if FDG uptake (SUV_max_) differed by 50% (e.g., means of 40 and 20 among nonresponders and responders, respectively) with a standard deviation no greater than 16, or a coefficient of variation no larger than 0.4. Measures of agreement, time to event models, and proportional odds models were considered exploratory, as the study generated estimates required to calculate study power and/or precision for these analyses in subsequent studies.

Thus, the study planned to enroll 42 patients, of whom 25 were expected to be evaluable. Evaluable patients were defined as those patients who completed cycle 1 of cetuximab and underwent pre- and post treatment CT and FDG-PET/CT. To accommodate one interim analysis and a final analysis while maintaining an overall 0.05 significance level, *P*-values were adjusted for multiple looks at the data using an O'Brien-Fleming test. The *P*-value for the first interim analysis was 0.0052 and for the final analysis 0.0480.

## Results

### Patient and tumor characteristics

Forty-two patients were enrolled onto the trial and 27 were evaluable for the objectives. All evaluable patients had SCCHN, underwent CT and FDG-PET/CT before and after cycle 1 of cetuximab, and were followed for the TTP endpoint. Of the 15 nonevaluable patients, eight developed rapidly progressive disease before week 8 of cycle 1, three developed a HSR prompting discontinuation of cetuximab, three had cutaneous SCC of the head and neck and were excluded, and one was deemed ineligible post hoc (target lesion <1.5 cm on FDG-PET/CT).

Most of the 27 evaluable patients were smokers with SCCHN that recurred within 1 year of primary therapy and had prior exposure to a platin agent (Table 1). Eight patients (29.6%) had prior exposure to cetuximab, given 12 or more months before study enrollment. Approximately half of the patients had received prior chemotherapy for recurrent disease. The characteristics of the evaluable patients and of all patients enrolled onto the trial were similar.

**Table 1:** Patient, tumor, and treatment characteristics.

Characteristic	Evaluable number (*n* = 27)	Percent
Patient		
Age (years) median (range)	63 (35–87)	–
Sex		
Male	21	77.8
Female	6	22.2
Race		
Caucasian	23	85.2
African-American	3	11.1
Asian	1	3.7
Performance status^1^		
0	5	18.5
1	13	48.1
2	9	33.3
Smoking history		
Yes	24	88.9
No	3	11.1
Interval from diagnosis to recurrence (months)		
Median (range)	10 (0–43)	–
Tumor		
Primary site		
Oral cavity	10	37.0
Oropharynx	8	29.6
Larynx	4	14.8
Hypopharynx	5	18.5
HPV-related oropharynx only	3/8	–
Prior treatment for initial disease		
Surgery		
Yes	15	55.6
No	12	44.4
Radiation^2^		
Yes	24	88.9
No	3	11.1
Chemotherapy^3^		
Yes	9	33.3
No	18	66.7
Cetuximab		
Yes	8	29.6
No	19	70.4
Interval from prior cetuximab treatment to recurrence (months)		
Median (range)	18.5 (12–48)	–
Prior treatment for recurrent disease		
Chemotherapy^4^		
Platin	9	33.3
Taxane	9	33.3
Pemetrexed or 5-FU	3	11.1
Targeted therapy^5^	3	11.1
# Lines of chemotherapy		
0	15	55.6
1	8	29.6
2–3	4	14.8
Prior platin exposure		
Yes	17	63
No	10	37

1 ECOG–Eastern Cooperative Oncology Group.

2 Postoperative or definitive.

3 Induction and/or chemoradiation.

4 Patients may have received one or more agents.

5 Vandetanib (2); Bevacizumab (1); Gefitinib (1).

### Primary objective

**Figure 1:**
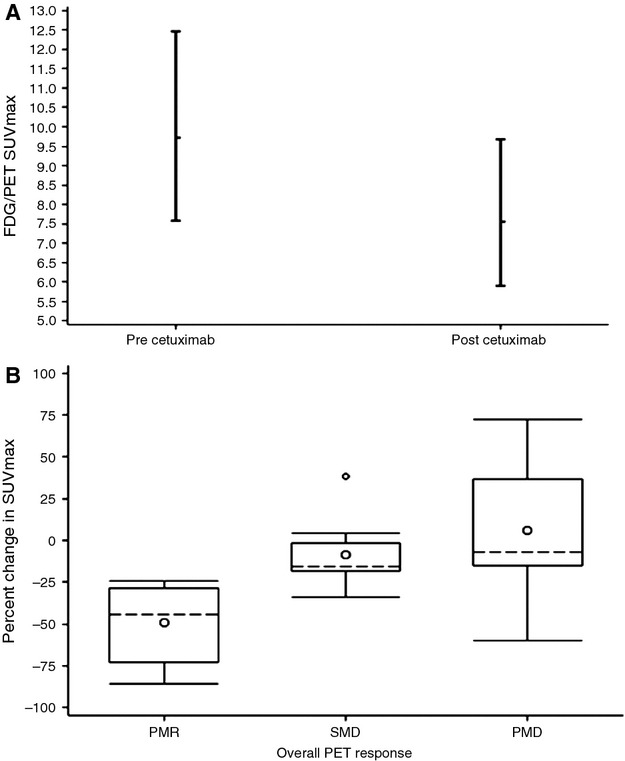
(A) Mean SUV_max_ pre and post cycle 1 of cetuximab adjusted for prior cetuximab (yes/no), study treatment is first-line cetuximab (yes/no) and tumor site (oropharynx/other). (B) Percent change in SUV_max_ pre and post cycle 1 of cetuximab by FDG-PET/CT response. SUV_max_, maximum standardized uptake value; FDG-PET/CT, F-18 fluorodeoxyglucose positron emission tomography/computed tomography; PMR, partial metabolic response; SMD, stable metabolic disease; PMD, progressive metabolic disease.

The primary objective was to compare the SUV_max_ of up to three target lesions as assessed by FDG-PET/CT before and then after 8 weeks of cetuximab. A linear hierarchical repeated measure model was used to estimate mean SUV on a log scale before and after cycle 1 of cetuximab. The model was adjusted for prior cetuximab exposure (yes/no), first-line versus subsequent-line cetuximab treatment (yes/no) and primary tumor site (oropharynx/other sites). The reference *P*-value determining statistical significance was. 048 to adjust for one interim analysis. Mean log SUV_max_ decreased with cycle 1 of cetuximab (*P* = 0.0097). On the original scale, SUV_max_ decreased by 21% from a precetuximab mean of 9.3 (95% confidence interval [CI]: 7.2, 12.1) to a post cycle 1 cetuximab mean of 7.3 (CI: 5.6, 9.5) (Fig. 1A). The percent change in SUV_max_ pre- and post cycle 1 of cetuximab grouped by metabolic tumor response is shown in Figure 1B.

### Overall anatomic and metabolic tumor response, and concordance of FDG-PET/CT and CT

Following cycle 1 of cetuximab, the overall anatomic tumor responses assessed by CT were PR/SD in 20 patients (74%) and progression in seven patients (26%). The overall metabolic tumor responses assessed by FDG-PET/CT were PMR/SMD in 15 patients (56%) and PMD in 12 patients (44%) (Table 2). A test for concordance found that FDG-PET/CT and CT tumor response assessments were discordant in 14 of the 27 patients (*P* = 0.0029) and had a low level of agreement (*κ* = 0.30 with 95% CI: 0.12, 0.48). A comparison of the two response variables illustrated the discordance in the form of lower disease control by FDG-PET/CT relative to CT. However, FDG-PET/CT was more likely to identify PR (10 of 27) than CT (1 of 27); whereas CT was more likely to identify patients as stable (19 of 27) than FDG-PET/CT (5 of 27).

**Table 2:** Concordance between CT and FDG-PET/CT after cycle 1 of cetuximab.

Response by CT at end of 1 cycle	Overall PET response			
	
Frequency	PMR	SMD	PMD	Total
PR	1	0	0	1
SD	9	5	5	19
PD	0	0	7	7
Total	10	5	12	27

FDG-PET/CT, F-18 fluorodeoxyglucose positron emission tomography/computed tomography; PMR, partial metabolic response; SMD, stable metabolic disease; PMD, progressive metabolic disease; PR, partial response; SD, stable disease; PD, progressive disease.

Mean percent changes of SUV_max_ of target lesions after cycle 1 were −48% (−24 to −81), −10% (0 to −17), and +8% (+72 to −57) when overall tumor responses by FDG-PET/CT were PMR, SMD, and PMD, respectively (Table 3). Two patients with ≥20% decrease in SUV_max_ of target lesions were classified as PMD because of interval increase in the number, FDG uptake, and/or size of nontarget lesions.

**Table 3:** Metabolic tumor response assessment by FDG-PET/CT for the 27 evaluable patients.

Overall response by FDG-PET/CT (*N* = 27)	Patient number	Number of target lesion(s)	SUV_max_			Nontarget lesion(s)^1^		
	
			Baseline	Post 8 weeks cetuximab	% Change	Number	SUV uptake	Size
Partial (37%)	10	1	14.4	10.3	−29	Stable	–	–
	15	1	16.6	12.1	−27	Stable	Stable	Stable
	16	1	8.6	5.7	−34	Stable	↓	Stable
	18	3	8.9	3.3	−63	↓	↓	↓
	19	1	14.8	4.0	−73	↓	↓	↓
	27	1	11.5	8.7	−24	↓	↓	↓
	32	2	3.3	2.1	−36	↓	↓	↓
	33	1	40.0	15.7	−61	–	–	–
	37	1	14.4	7.4	−49	–	–	–
	39	1	8.3	1.6	−81	Stable	Stable	Stable
*N* = 10	Mean of column	11 (1–3)	14.1 (3.3–40.0)	7.1 (1.6–15.7)	−48 (−24 to −81)			
Stable (19%)	5	3	7.5	7.5	0	Stable	Stable	Stable
	12	1	5.4	4.5	−17	Stable	Stable	Stable
	22	1	6.5	6.0	−8	Stable	Stable	Stable
	24	2	20.6	18.1	−12	–	–	–
	28	1	12.7	10.8	−15	Stable	Stable	Stable
*N* = 5	Mean of column	2 (1–3)	10.5 (5.4–20.6)	9.4 (4.5–18.1)	−10 (0 to −17)			
Progression (44%)	1	1	5.7	8.2	+44	–	–	–
	2	1	8.9	7.4	−17	Stable	↑	↑
	8	3	8	8	0	↑	↑	Stable
	20	3	3.8	5.5	+45	↑	↑	↑
	21	1	20.2	8.6	−57	↑	↑	↑
	23	3	14.0	19.8	+41	↑	↑	↑
	26	1	12.6	15.4	+22	Stable	↑	↑
	31	3	14.8	12.9	−13	Stable	Stable	↑
	34	1	18.7	17.9	−4	↑	↑	↑
	35	3	14.2	10.4	−27	↑	↑	↑
	40	1	5.4	9.3	+72	↑	↑	Stable
	42	3	10.1	9.0	−11	↑	↑	↑
*N* = 12	Mean of column	2 (1–3)	11.4 (3.8–20.2)	11.0 (5.5–19.8)	+8 (+72 to −57)			

FDG-PET/CT, F-18 fluorodeoxyglucose positron emission tomography/computed tomography; SUV_max_, maximum standardized uptake value.

1 –, Not applicable.

### Agreement in treatment decision between CT and FDG-PET/CT

**Figure 2:**
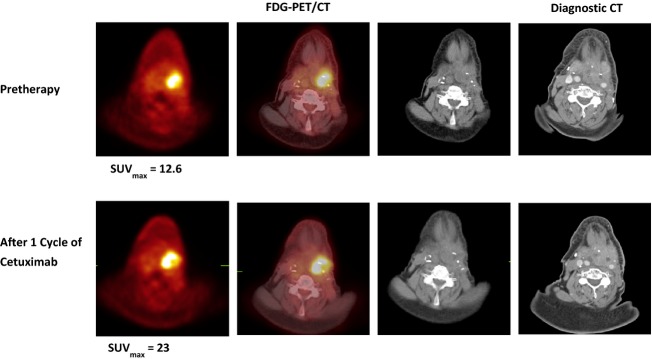
Discordant CT and FDG-PET/CT in a patient (#26) with recurrent left hypopharyngeal squamous cell carcinoma. Transaxial FDG-PET/CT (PET, left; fused PET/CT, middle; CT, right) and contrast-enhanced diagnostic CT images pretherapy (upper row) and after 1 cycle of cetuximab (lower row). The SUV_max_ within the recurrent left hypopharyngeal mass increased from 12.6 pretherapy to 23 after 1 cycle of cetuximab, indicating progression, but the size of the mass was stable on the diagnostic CT scan. SUV_max_, maximum standardized uptake value; FDG-PET/CT, F-18 fluorodeoxyglucose positron emission tomography/computed tomography.

We assessed the agreement in treatment decision based on the tumor response assessment by CT and FDG-PET/CT following cycle 1 of cetuximab. For study purposes, agreement in treatment decision was defined to occur when tumor response assessment by CT and FDG-PET/CT would have resulted in the same decision to either continue cetuximab (if disease control) or to stop cetuximab (if progression). Conversely, disagreement in treatment decision was defined to occur when tumor response assessment by CT and FDG-PET/CT would have resulted in different treatment decisions. Using these clinically relevant definitions, we observed agreement in treatment decision between the two imaging modalities after cycle 1 in 22 patients (81.4%) and disagreement in treatment decision between the two imaging modalities in five patients (18.6%) (Table 2). All five cases of disagreement in treatment decision showed stable anatomic response by CT and PMD by FDG-PET/CT. An example of disagreement in treatment decision between the two imaging modalities is shown in Figure 2.

### Tumor response and TTP

Cetuximab was continued after cycle 1 in patients with disease control (PR/SD) by CT, even if the FDG-PET/CT showed PMD. Cetuximab was discontinued after cycle 1 in patients with progression by CT. In the 20 evaluable patients with disease control by CT after cycle 1, median TTP was 166 days (CI: 86, 217) if the FDG-PET/CT showed disease control (*n* = 15) and 105 days (CI: 66, 159) if the FDG-PET/CT showed progression (*n* = 5) (*P* < 0.0001). All seven patients with progression by CT after cycle 1 also had PMD by FDG-PET/CT. The median TTP in these patients was 53 days (CI: 49, 56).

We compared the TTP in each tumor response group between the two imaging modalities. Median TTP of the 20 patients whose post cycle 1 tumor response assessment showed disease control by CT compared to the 15 patients whose post cycle 1 tumor response assessment showed disease control by FDG-PET/CT were 113 (CI: 93, 167) versus 166 (CI: 86, 217) days, respectively. Median TTP of the seven patients whose post cycle 1 tumor response assessment showed progression by CT compared to the 12 patients whose post cycle 1 tumor response assessment showed PMD by FDG-PET/CT were 53 (CI: 49, 56) versus 61 (CI: 50, 105) days, respectively.

## Discussion

This is the first prospective report to describe metabolic tumor response assessment by FDG-PET/CT to single-agent cetuximab in patients with incurable SCCHN and to compare these results to anatomic tumor response assessment by CT. Tumor response assessment by CT is a poor method of determining benefit from cetuximab in patients with incurable SCCHN, as judged by the disparity between a high disease control rate (46%) and the short TTP (70 days) [1]. We determined agreement in treatment decision using a clinically relevant definition: tumor response assessment by CT and FDG-PET/CT resulted in the same decision to either continue cetuximab (if disease control) or to stop cetuximab (if progression). Disagreement in treatment decision between CT and FDG-PET/CT would in theory lead to different decisions about continuation of cetuximab following cycle 1. We investigated whether TTP differed in patients with disease control (PR/SD) by CT after cycle 1 based on the agreement/disagreement with FDG-PET/CT. Note that cetuximab was continued after cycle 1 in patients with disease control by CT, even if the FDG-PET/CT showed PMD. Cetuximab was discontinued after cycle 1 with progression by CT. In the 20 patients with disease control by CT after cycle 1 of cetuximab, median TTP was 166 days (CI: 86, 217) if the FDG-PET/CT showed disease control and 105 days (CI: 66, 159) if the FDG-PET/CT showed progression (*P* < 0.0001). These data suggest that patients with disease control by CT following cycle 1 of cetuximab but with progression by FDG-PET/CT (25% of this group) are benefitting less from the cetuximab in comparison to those in whom there is agreement in the two imaging modalities, and thus, may be candidates for alternative therapy.

We stratified patients based on the agreement between the two imaging modalities in either disease control or progression after cycle 1 of cetuximab and then compared TTP between the two imaging modalities in each tumor response group. Median TTP of the 20 patients whose post cycle 1 tumor response assessment showed disease control by CT compared to the 15 patients with disease control by FDG-PET/CT were different (109 [CI: 93, 167] vs. 166 [CI: 86, 217] days, respectively). Median TTP of the patients whose post cycle 1 tumor response assessment showed progression by CT compared to the patients with PMD by FDG-PET/CT were similar (53 [CI: 49, 56] vs. 61 [CI: 50, 105] days, respectively). These data suggest that FDG-PET/CT is a better predictor of TTP than CT for patients with disease control after cycle 1 of cetuximab.

The primary objective of this trial was to compare the SUV_max_ of target lesions as assessed by FDG-PET/CT before and after 8 weeks of cetuximab. The mean percent change of SUV_max_ of target lesion(s) after cycle 1 was −21% (range: +72% to −81%) for the 27 evaluable patients. The change in mean SUV_max_ of the target lesions after cycle 1 was ≥20% decrease in 12 patients, ≥20% increase in five patients, and between these parameters in 10 patients. For comparison, one study observed >25% decrease in SUV_max_ of target lesion(s) in 18 of 19 patients after 2 weeks of cetuximab given preoperatively to treatment-naïve SCCHN patients scheduled for primary curative surgery [5]. However, another smaller trial observed a mean percent decrease in SUV_max_ of target lesion(s) of only −11.1% (range 0 to −24.4) after 2 weeks of cetuximab given before radiation therapy and concurrent cetuximab [12].

Interestingly, a ≥10% decrease in SUV_max_ of the target lesion(s) in association with an increase in the number, SUV and/or size of the nontarget lesions consistent with overall PMD by FDG-PET/CT occurred in five patients (19%) in our study. This observation may reflect the heterogeneity of metastatic tumor deposits within a patient such that one lesion may be responsive to cetuximab, whereas another lesion(s) may be resistant. Genetic diversity of metastatic tumor deposits within a patient may be the mechanism of this observation [13].

Studies in other malignancies responsive to EGFR inhibitors have examined the utility of FDG-PET/CT. In advanced nonsmall cell lung cancer (NSCLC), FDG-PET/CT was found to be a predictor of nonprogression (measured by CT), progression-free survival, and OS with erlotinib therapy [14]. Patients with mutated EGFR compared to wild-type EGFR were found to have a greater percent reduction in SUV_peak_ with erlotinib. These data link proportional reduction in SUV_peak_ with benefit of single-agent EGFR inhibition in NSCLC, analogous to our observations with EGFR inhibition by cetuximab in incurable SCCHN. Other preclinical [15] and clinical studies [16–18] showed a correlation of tumor response to EGFR inhibition in NSCLC to early reduction in SUV as assessed by FDG-PET/CT, particularly in tumors with EGFR mutations.

Limitations of our trial include the small number of evaluable patients, absence of randomization to continue cetuximab or change therapy based on the tumor response to cycle 1 assessed by CT or FDG-PET/CT, and single-institution experience. FDG-PET/CT was performed without IV iodine contrast; however, two recent surveys found that there was wide variability in whether IV contrast was administered with FDG-PET/CT [19, 20]. The majority of facilities perform noncontrast-enhanced FDG-PET/CT. It is also possible that PET with radiotracers that measure other functional tumor characteristics such as proliferation (^18^F-fluorothymidine) may be complementary to or better than FDG-PET/CT in assessing tumor response to cetuximab [21]. Several patients developed progressive disease before completing cycle 1, suggesting that FDG-PET/CT may be best tested earlier in treatment or that FDG-PET/CT may have had no role in these patients. However, our study is an important step forward to address an unmet need in the development of methods that allow for more accurate assessment of benefit of cetuximab in patients with SCCHN.

The limited number of evaluable patients in our trial precludes firm conclusions; however, several observations generate testable hypotheses. In this prospective trial, we show that FDG-PET/CT may be better than CT in assessing benefit of single-agent cetuximab in patients with incurable SCCHN. Patients with disease control by CT following cycle 1 of cetuximab but with progression by FDG-PET/CT (25% of this group) are benefitting less so or not at all from the cetuximab. In addition, FDG-PET/CT was a better predictor of TTP than CT in patients with disease control as assessed by CT after cycle 1 of cetuximab. It is important to develop methods that more accurately assess efficacy of cetuximab in patients with incurable SCCHN since this costly targeted agent benefits a minority of patients. These observations provide evidence to perform a controlled trial to validate the findings.
